# Effects of meteorology and lunar cycle on the post-thawing quality of avian sperm

**DOI:** 10.3389/fvets.2024.1394004

**Published:** 2024-05-16

**Authors:** Esther Díaz Ruiz, Juan Vicente Delgado Bermejo, Antonio González Ariza, José Manuel León Jurado, Ander Arando Arbulu, Francisco Javier Navas González

**Affiliations:** ^1^Department of Genetics, Faculty of Veterinary Sciences, University of Córdoba, Córdoba, Spain; ^2^Agropecuary Provincial Centre, Diputación Provincial de Córdoba, Córdoba, Spain

**Keywords:** avian reproduction, climatic condition, lunar cycle, endangered breed, motility, sperm cryopreservation, sperm freezability

## Abstract

**Introduction:**

Various climatological and lunar cycle parameters have a direct impact on animal reproduction, and in the case of the avian species, spermatozoa are extremely sensitive to heat stress. These parameters could influence sperm freezability, which will ultimately affect post-thawing semen quality, being sperm motility in roosters a relevant indicator of this quality as it is highly related to fertility. Therefore, the objective of the present study is to determine which are the climatological and lunar cycle parameters that have a greater effect on sperm freezability in roosters.

**Methods:**

Sperm was obtained from 16 Utrerana breed roosters and a total of 27 replicates were performed. A pool was made with those ejaculates that met the minimum quality criteria for each replicate, and four freezing–thawing samples per replicate were analyzed. The straws were thawed, and sperm motility was evaluated, classifying the results obtained into four seminal quality groups according to the guidelines of the Food and Agriculture Organization of the United Nations (Group 1: Good, Group 2: Satisfactory, Group 3: Acceptable but undesirable and Group 4: Unsatisfactory). The following traits were recorded for each day of semen collection: maximum temperature, minimum temperature, maximum barometric pressure, minimum barometric pressure, maximum gust, wind direction, mean wind speed, sunshine hours, rainfall, moon phase, and percentage of illuminated lunar surface over the total area.

**Results:**

A discriminant canonical analysis was performed to determine which of these parameters offered the most information when classifying an ejaculate in each quality group, with minimum temperature, the new moon as moon phase, minimum barometric pressure, and rainfall being the most significant variables.

**Discussion:**

According to the results obtained, semen quality decreases when temperature and precipitation are lower, pressure is higher, and when there is a new moon phase. Therefore, these environmental conditions should be avoided for sperm collection and processing.

## 1 Introduction

Climatic conditions have an impact on animal reproduction, which is reflected in the different reproductive parameters. Temperature, humidity, and atmospheric pressure have been reported to be important factors that determine the seminal quality of roosters (1, 2). Heat stress generates numerous reactive oxygen species (ROS) which causes an oxidative imbalance that is more accentuated in the avian species due to the high amount of polyunsaturated fatty acids (PUFAs) present in avian spermatozoa which are more prone to lipid peroxidation in the presence of ROS (3, 4). In addition, a high temperature is associated with lower sperm concentration and motility (5). The lunar cycle is another factor that also influences animal reproduction, causing fluctuations in corticosterone and melatonin levels in birds (6).

Since multiple factors can influence reproductive parameters, in males, the seminal quality evaluation of ejaculates is important and can be done by different methods. There are tools to evaluate both macroscopic parameters, such as ejaculate volume and color, as well as microscopic parameters such as concentration, morphology, or sperm motility (7). In the case of birds, sperm motility is a very relevant factor since it is highly related to fertility because only those spermatozoa with a good motility level will be able to ascend through the oviduct to reach the fertilization zone (8).

Within assisted reproduction techniques, sperm cryopreservation is a very valuable tool for the conservation of genetic resources in avian species (9). However, sperm viability is generally reduced by 30–60% after a freeze–thaw cycle (8, 10). A factor that can influence sperm freezability is the season, since it causes changes in the biochemical composition of the ejaculate, resulting in more favorable motility in thawed semen when the seminal collection is performed in spring, as has been reported in local Spanish poultry breeds kept in natural environmental conditions (11).

Seminal cryopreservation techniques must be improved in poultry species. Avian germplasm banks are very incomplete, compared to those of other livestock species (12), since poultry semen is more vulnerable due to specific characteristics of avian spermatozoa (13). This is due mainly to the physiological and anatomical peculiarities of birds, such as the intra-abdominal placement of testes and the absence of accessory sex glands (13). Thus, a technical and research effort must be made by public institutions for the development of techniques and tools to improve *in vitro* conservation of local genetic resources.

A bank of a local genotype should be considered complete when the stored genetic material allows the reconstruction of the breed, if necessary, as well as the increasing the effective size of a population by reducing genetic drift, among others (14). An example of an endangered local breed is the Utrerana avian breed, whose census as of 31/12/2022 did not exceed 2000 individuals (12) This breed belongs to the Mediterranean trunk and shows high rusticity, which allows its adaptability to more sustainable extensive production systems with minimum impact on the environment. Thus, the conservation of this type of breed has an indirect positive impact on human health and its breeding systems are also more respectful of animal welfare (15, 16). Utrerana hen was initially oriented toward egg production, however, the introduction of rather productive commercial hybrid genotypes in Europe produced the displacement of this breed to an endangered position (17). Given its current complicated situation in terms of the existing census of the breed, the development of a germplasm bank for the breed should be an important tool for the successful implementation of its conservation program.

In any case, the control of climatological parameters could bring benefits to the cryopreservation process, so the main objective of the present research is to determine the existing relationships between the different climatic parameters and the lunar cycle on the post-thawing seminal quality of roosters’ ejaculates concerning sperm motility.

## 2 Materials and methods

### 2.1 Ethical approval

The present study is excluded from the scope of evaluation of the Ethics Committee of the University of Córdoba since data obtained are part of routine activities carried out at the Agropecuary Provincial Center of the Diputación of Córdoba (Andalusia, Spain) as a center for avian reproduction and conservation of native breeds and therefore are not considered animals used for scientific purposes. However, all animals used in the study were treated following European Legislation (Directive 2010/63/EU “on the protection of animals used for scientific purposes”), which has been transposed into Spanish law through RD 53/2013.

### 2.2 Animal sample

For this study, 16 Utrerana roosters breed between 1 and 3 years of age were housed in individual cages (95 × 95 × 95 cm) in the Agropecuary Provincial Center of the Diputación of Córdoba (37°54′50.9”N-4°42′40.4”W, Andalusia, southern Spain) under a natural photoperiod. All the animals were fed a commercial diet (15.20% crude protein, 4.60% crude fat and oils, 3.20% crude fiber, 14.00% crude ash, 4.10% calcium, 0.66% phosphorus, 0.19% sodium, 0.31% methionine, 0.72% lysine) and water was supplied *ad libitum*.

### 2.3 Semen collection and processing

Semen collection was carried out using the dorsal-abdominal massage technique described by Burrows and Quinn (18). Semen collection was carried out between September 2021 and May 2022. For each working day, a pool was made with those samples that met previously established minimum quality criteria: volume (>0.2 mL), concentration (>3×10^9^ spz/mL), motility (≥80%), and morphology (≤10 to 15%). A total of 27 replicates were performed, analyzing four samples per replicate. Table 1 shows the number of samples used for each season and phase of the lunar cycle.

**Table 1:** Numbers of samples used for each season and phase of the lunar cycle.

Number of samples used for each season	Summer	4
	Autumn	36
	Winter	24
	Spring	44
Number of samples used for each lunar cycle	Full moon	4
	Waning gibbous	24
	Waning crescent	24
	New moon	4
	Waxing crescent	24
	Waxing gibbous	28

Each semen sample was refrigerated in a programmable cooler (Cell incubator SH-020S, Welson, Korea) for 1 h until it reached 5°C with a temperature decrease rate of 0.3°C/min. After that hour the semen was diluted with a diluent which was composed by 0.2 g D (+)-glucose, 3.8 g D (+)-trehalose dihydrate, 1.2 g L-glutamic acid monosodium salt, 0.3 g potassium acetate, 0. 08 g magnesium acetate tetrahydrate, 0.05 g sodium citrate tri-basic dihydrate, 0.4 g BES, 0.4 g Bis-Tris and 0.001 g gentamicin sulfate (pH = 6.8, osmolarity = 360 mOsm) (19). After 30 min, a second dilution was carried out with the diluent described above, to which N-methylacetamide (NMA) was added as a cryoprotectant at a concentration of 18% (final concentration of 9%). After the second dilution, the sperm were packed in 0.25 straws at a final concentration of 250 × 10^6^ spz/straw and 30 min later placed in nitrogen vapors at a height of 4 cm for 30 min and finally immersed in liquid nitrogen (−196°C).

### 2.4 Sperm motility assessment

For the evaluation of sperm motility, four straws per replicate (a total of 108 observations) were thawed by immersing each straw in a 5°C water bath for 1 min 40 s (19). Total motility (TM, %) was analyzed using a Computer Assisted Sperm Analyser IVOS 12.3 (Hamilton-Thorne Bioscience, MA, United States). For this purpose, 5 μL of the sample was deposited in a Life Optic chamber. This sample had been previously diluted to 50 × 10^6^ spz/mL with the diluent described above, considering as spermatozoa those cells with an area between 2 and 60 μm^2^.

To classify the samples analyzed, four groups were established according to the quality of post-thawed sperm concerning TM based on the criteria published by the Food and Agriculture Organization of the United Nations (FAO) (20), which are as follows: Group 1: Good (>50%), Group 2: Satisfactory (40–50%), Group 3: Acceptable but undesirable (30–40%) and Group 4: Unsatisfactory (<30%).

### 2.5 Data collection: climatic parameters and lunar cycle

For each day of seminal extraction, the environmental conditions in the Agropecuary Provincial Center of the Diputación of Córdoba were analyzed for different climatological parameters such as maximum temperature, minimum temperature, maximum barometric pressure, minimum barometric pressure, maximum gust, wind direction, mean wind speed, sunshine hours and rainfall, and the lunar cycle such as moon phase and percentage of illuminated lunar surface over the total area. The minimum and maximum values for each parameter are presented in Table 2. The data for the climatological parameters were obtained from the State Meteorological Agency^1^ and those for the lunar cycle from the web page of the Astronomical Applications Department of the United States Naval Observatory.^2^

**Table 2:** Minimum and maximum values for the parameters evaluated.

Variable	Minimum level	Maximum level
Maximum temperature (°C)	16.7	40.6
Minimum temperature (°C)	0.8	18.4
Maximum barometric pressure (mb)	1001.2	1018.6
Minimum barometric pressure (mb)	994.6	1013.3
Maximum gust speed (m/s)	4.7	18.1
Wind direction	4	99
Mean wind speed (m/s)	0.8	5.6
Sunshine hours	3.8	14
Rainfall (l/m2)	0	7.8
Moon phase		
Filling of the moon (%)	0	100

### 2.6 Overall descriptive statistics

The mean of each semen quality group was established for the meteorological and lunar cycle traits registered: maximum temperature, minimum temperature, maximum barometric pressure, minimum barometric pressure, maximum gust speed, wind direction, mean wind speed, sunshine hours, rainfall, moon phase and percentage of moon filling. To perform this analysis, we used the descriptive statistics routine of the data description package of XLSTAT software (Addinsoft Pearson Edition 2014, Addinsoft, Paris, France).

### 2.7 Statistical analysis: discriminant canonical analysis

For the DCA, the 11 explanatory variables regarding the meteorological and lunar cycle traits mentioned above were included. The classification criteria established correspond to the four semen quality groups mentioned previously (1: good, 2: satisfactory, 3: acceptable but undesirable, and 4: unsatisfactory) and the differences between groups were measured.

According to several authors, a minimum sample size of at least 20 observations per each of 4 or 5 predictors is required, and the maximum number of independent variables to mitigate possible distortion effects should be n-2, where n is the sample size. Taking this into account, the sample size used in this study would be correct (21, 22).

Multicollinearity analysis ensures the existence of strong and independent relationships between predictors. Among the forward selection methods, the forward method was carried out because it requires less execution time (17). To perform multicollinearity and DCA analyses, the Discriminant Analysis routine of the Analyzing Data package of XLSTAT software (Addinsoft Pearson Edition 2014, Addinsoft, Paris, France) was used.

#### 2.7.1 Multicollinearity preliminary testing

Before performing a DCA, the assumption of multicollinearity was checked to ensure that the variance of the explanatory potential is not over-inflated by problems of redundancy between variables. The variance inflation factor (VIF) is the most widely used indicator for detecting multicollinearity, and previous authors have recommended values lower than 5 to discard redundancy problems (23). VIF was computed by using the following formula as a subroutine of the Discriminant Analysis routine of the Analyzing Data package of XLSTAT software:


$$VIF=1/\left(1-R^{2}\right),$$

where *R*^2^ was the coefficient of determination of the regression equation.

#### 2.7.2 DCA model reliability

When DCA is used and there are unequal sample sizes, the only acceptable test that can be applied is the Pillai’s trace criterion. The assumption of equal covariance matrices was tested through this method in the discriminant function analysis (24). A subroutine of the Discriminant Analysis routine of the Analyzing Data package of XLSTAT software was used to calculate this parameter, considering the set of predictors of the DCA statistically significant when *p* ≤ 0.05. Pillai trace criterion has been argued to be the most robust statistic for general protection against deviations from normality and homogeneity of variance of multivariate residuals. The evidence that the set of predictors has a statistically significant effect on the values of the response variable increases as the value of the Pillai trace is higher.

#### 2.7.3 DCA efficiency

The contributions of the variables to the discriminant function were analyzed through the use of Wilks’ Lambda test. The contribution is greater as the Wilks lambda value approaches 0. The functions can be used to explain group adscription if *p* ≤ 0.05 (25).

#### 2.7.4 Independent factor discriminant potential evaluation, canonical coefficients, and loading interpretation

After analyzing the variables whose discriminant potential was based on the differences in means between the different treatments, a discriminant function analysis was carried out to identify those whose discriminant potential could be based on their ability to determine higher percentages of assignment of observations within their group. Discriminant values of ≥ |0.40| indicate that the discriminant load of the different variables is significantly discriminant. To avoid the inclusion of redundant variables in the function, a stepwise procedure technique is used. A higher discrimination capacity and a higher percentage of correct classification were obtained when the absolute values of the loadings of the standardized coefficients of each variable were high.

#### 2.7.5 Spatial representation

Squared Mahalanobis distances and principal component analysis were computed, using the following formula:


$$D_{ij}^{2}=\left({\bar{ϒ}}_{i}-{\bar{ϒ}}_{j}\right)\;COV^{-1}\left({\bar{ϒ}}_{i}-{\bar{ϒ}}_{j}\right),$$

where $D_{ij}^{2}$ : distance between population i and j; COV^−1^: inverse of the covariance matrix of measured variable x; ${\bar{ϒ}}_{i}$ and ${\bar{ϒ}}_{j}$ : means of variable x in the ith and jth populations, respectively (25).

A dendrogram was constructed by converting the squared Mahalanobis distance matrix into a Euclidean distance matrix. For this, the underweight pair-group method arithmetic averages (UPGMA; Rovira i Virgili University, Tarragona, Spain), and the Phylogeny procedure of MEGA X 10.0.5 (Institute of Molecular Evolutionary Genetics, The Pennsylvania State University, State College, PA, United States) were used.

#### 2.7.6 Discriminant function cross-validation

The probability that an observation of an unknown background is correctly classified in a given group can be determined by calculating the hit ratio (26). To determine whether the different discriminant functions can be validated, the leave-one-out cross-validation option was used (27). When the classification rate is at least 25% higher than that obtained by chance, DCA can be considered to achieve classification accuracy.

These results obtained must be supported by Press’ Q statistic, which is a parameter that can compare the discriminating power of the cross-validated function by using the formula:


$$\mathrm{Pres}s^{\prime }\;Q={\left[n-\left(n^{\prime }K\right)\right]}^{2}/\left[n\;\left(K-1\right)\right],$$

where n is the number of observations in the sample; n’ is the number of observations correctly classified, and K is the number of groups.

The value of Press’ Q statistic should be compared to the critical value of 6.63 for *χ*^2^ with a degree of freedom in a significance level of 0.01. To consider the cross-validated classification to be significantly better than chance, the value of Press’ Q must exceed the critical value of *χ*^2^ = 6.63.

## 3 Results

### 3.1 Overall descriptive statistics

The mean for each meteorology or lunar cycle-related trait in each seminal quality-related group is shown in Table 3. The highest values for minimum temperature (13.205) were reported for group 1. On the other hand, the lowest values for this trait were reported in group 4 (6.400). All samples made in the new moon lunar cycle were reported to belong to group 4. Lastly, samples obtained in group 4 obtained the highest values for minimum barometric pressure (1007.433).

**Table 3:** Means by class; n: sample size for each group; MT values [expressed as mean (SD)] for each group were as follows: 1 = 57.75 (7.38), 2 = 44.48 (3.21), 3 = 34.59 (3.00), and 4 = 20.62 (6.14).

Group	1 (*n* = 20)	2 (*n* = 40)	3 (*n* = 27)	4 (*n* = 21)
Minimum temperature	13.205	10.130	11.107	6.400
Minimum barometric pressure	1004.275	1003.095	1005.459	1007.433
Wind direction	38.000	39.550	44.222	29.333
Average wind speed	2.420	2.765	2.326	2.067
Sunshine hours	9.520	8.918	10.326	9.738
Rainfall	1.440	0.995	0.041	0.033
Moon phase-full moon	0.050	0.050	0.037	0.000
Moon phase-new moon	0.000	0.000	0.000	0.190
Moon phase-waning crescent	0.450	0.225	0.148	0.095
Moon phase-waning gibbous	0.350	0.200	0.148	0.238
Moon phase-waxing crescent	0.100	0.250	0.222	0.286
Moon phase-waxing gibbous	0.050	0.275	0.444	0.190

### 3.2 DCA model reliability and efficiency

Maximum barometric pressure, maximum temperature, percentage of moon filling, and maximum gust speed were discarded for the following analyses as these variables showed a VIF value greater than 5. The variables that remained after the preliminary multicollinearity analysis (VIF < 5) are shown in Table 4.

**Table 4:** Variables that remained at preliminary multicollinearity analysis using variance inflation factor (VIF) of explanatory variables.

	Tolerance (1-*R*^2^)	VIF
Rainfall	0.418	2.392
Minimum barometric pressure	0.449	2.227
Sunshine hours	0.450	2.224
Moon phase-waning gibbous	0.457	2.190
Minimum temperature	0.508	1.968
Average wind speed	0.531	1.884
Moon phase-waxing crescent	0.557	1.797
Moon phase-waning crescent	0.557	1.797
Wind direction	0.653	1.532
Moon phase-new moon	0.728	1.374
Moon phase-full moon	0.756	1.323

**Figure 1:**
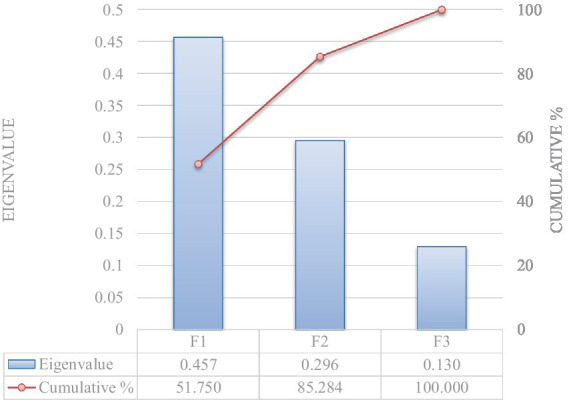
Eigenvalue and cumulative variability explanatory potential of independent explanatory variables.

Significant Pillai’s trace criterion determined the validity of the DCA (*p* < 0.0001; Table 5). Of the three discriminant functions revealed after discriminant analyses, two showed a significant discriminant ability (Significance of 0.010 and 0.041 for F1 and F2, respectively; Table 6). The discriminatory power of the function F1 was high (eigenvalue of 0.457) with 85.28% of the variance being explained by F1 and F2 (Figure 1).

**Table 5:** Summary of the results of pillai’s trace of equality of covariance matrices of canonical discriminant functions.

Trace	F (Observed value)	F (Critical value)	DF1	DF2	*p*-value	Alpha
0.657	2.446	1.477	33	288	<0.0001	0.05

**Table 6:** Discriminant canonical analysis efficiency parameters to determine the significance of each canonical discriminant function.

Test of Function(s)	Wilks’ lambda	Chi-square	Df	Sig.
1 through 3	0.700	34.906	18	0.010
2 through 3	0.843	16.704	10	0.041
3	0.962	3.831	4	0.429

### 3.3 Independent factor discriminant potential evaluation, canonical coefficients, and loading interpretation

The discriminating ability of the different variables studied is shown in Table 7. The greater discriminating power of a variable in question is related to a high value of F and consequently, lower values of Wilks’ Lambda, which translates into a better position in the rank. The present analysis revealed that minimum temperature (Wilks’ Lambda = 0.803; *F* = 8.504), new moon as moon phase (Wilks’ Lambda = 0.841; *F* = 6.571), minimum barometric pressure (Wilks’ Lambda = 0.886; *F* = 4.476), rainfall (Wilks’ Lambda = 0.897; *F* = 3.988), waxing gibbous (Wilks’ Lambda = 0.908; *F* = 3.518), and waning crescent (Wilks’ Lambda = 0.918; *F* = 3.084) as moon phases contributed significantly (*p* < 0.05) to the discriminant ability of significant discriminant functions.

**Table 7:** Results for the tests of equality of group means to test for difference in the means across sample groups once redundant variables have been removed.

Variable	Rank	Wilks’ Lambda	F	DF1	DF2	*p*-value
Minimum temperature	1	0.803	8.504	3	104	<0.0001
Moon phase-New Moon	2	0.841	6.571	3	104	0.000
Minimum barometric pressure	3	0.886	4.476	3	104	0.005
Rainfall	4	0.897	3.988	3	104	0.010
Moon phase-Waxing Gibbous	5	0.908	3.518	3	104	0.018
Moon phase-Waning Crescent	6	0.918	3.084	3	104	0.031
Average wind speed	7	0.950	1.823	3	104	0.147
Sunshine hours	8	0.955	1.619	3	104	0.190
Moon phase-Waxing Crescent	9	0.978	0.787	3	104	0.504
Wind direction	10	0.982	0.653	3	104	0.583
Moon phase-Full Moon	11	0.990	0.354	3	104	0.787

**Figure 2:**
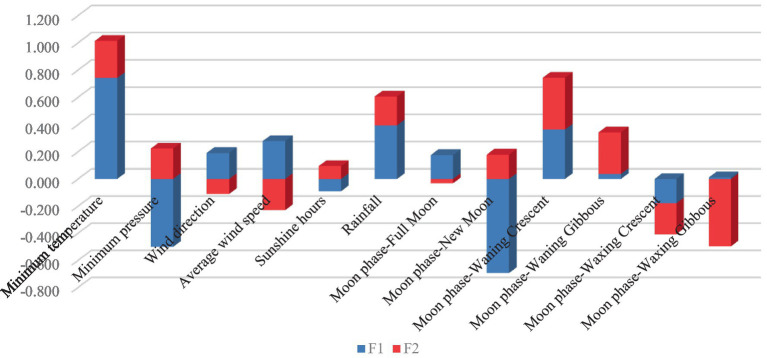
Standardized canonical discriminant function coefficients.

Figure 2 reports discriminant canonical coefficient loadings for representative variables across discriminant functions. Minimum temperature (coefficient value = │0.741│), new moon (coefficient value = │0.693│), and minimum pressure (coefficient value = │0.499│) were the traits with the highest standardized canonical discriminant function coefficients for F1. Waxing gibbous (coefficient value = │0.496│), waning crescent (coefficient value = │0.379│), and waning gibbous (coefficient value = │0.306│) were the traits that showed the highest coefficient values for F2.

### 3.4 Spatial representation

**Figure 3:**
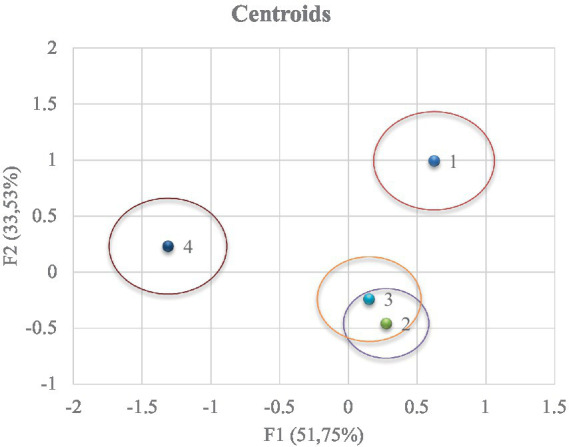
Territorial map depicting the results of the canonical discriminant analysis on the different groups.

A clear differentiation between treatments is observed (Figure 3). To obtain the coordinates of the x and y axes, the mean value of the observations in each term of the first two discriminant functions (F1 and F2) is substituted to obtain the relative position of the centroids. The predictive power of the canonical discriminant function to classify the observations will be higher as the distance between the centroids increases.

**Figure 4:**
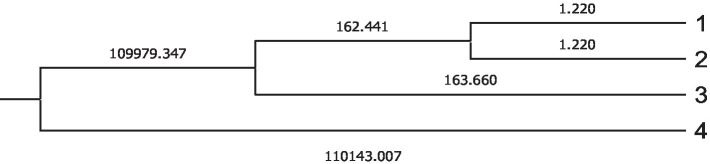
Cladogram constructed from Mahalanobis distances across different groups.

The Mahalanobis distance is calculated by the relative distance of the problem observation to the centroid of its nearest group representing the probability that an observation showing an unknown background belongs to a given group, and it is necessary to calculate the hit ratio which is shown in Figure 4. In both figures (Figures 3, 4), group 4 was reported to be obtained in meteorological and moon phase conditions that differed to a large extent from the rest of the groups.

### 3.5 Discriminant function cross-validation

A Press’ Q value of 215.11 (*n* = 108; *n*’ = 93; K = 4) was computed. Thus, predictions can be considered to be better than chance at 95%. Table 8 shows the cross-validation of discriminant classification results. The discriminant tool developed in the present study allowed to correctly classify 86.11% of the studied samples.

**Table 8:** Cross-validation of classification results.

From\To	1	2	3	4	Total	% Correct
1	20	0	0	0	20	100.00%
2	3	26	8	3	40	65.00%
3	0	0	26	1	27	96.30%
4	0	0	0	21	21	100.00%
Total	23	26	34	25	108	86.11%

## 4 Discussion

This research analyzes a series of climatic and lunar cycle traits. The verification of relationships between the explanatory variables is useful for deciding which combination of them makes up an effective predictive model. For this, the selection of those independent variables that do not overlap in terms of their ability to explain the variability of the data was necessary. Thus, variables with multicollinearity problems (VIF > 5) were not taken into account in the following analyses.

Maximum barometric pressure and maximum temperature were discarded due to multicollinearity problems with minimum barometric pressure and minimum temperature, respectively. In the atmosphere, elements such as temperature and barometric pressure show diurnal variations, which can be explained by the combination of multiple factors, with the solar component being the determining factor, and maximum and minimum temperatures occurring at approximately the same local time each day (28, 29). The daily temperature curve can be approximated by a sinusoidal function but can be influenced by latitude, time of year, and other climatic factors. However, factors such as clouds or wind can modify the curve temporally (30).

On the other hand, multicollinearity problems with the moon filling percentage could be related to the redundancy with the moon phase variable since both parameters are directly related. The phases of the moon are determined based on the illuminated portion that can be observed from the Earth as the moon moves around it (31). Moon phase could have an impact on animal physiology, as it influences cyclic changes in water, which constitutes 65–70% of birds (32, 33). In addition, previous authors have hypothesized that animals may be influenced by the moon due to changes in geomagnetic fields by increasing sensitivity to magnetoreception with a full moon (34). The influence of the lunar cycle on animal reproduction has been studied in several species such as cattle (35), pigs (36) or horses (37), however, a lack of knowledge of avian species in this field is evident.

Finally, the maximum gust speed variable, which could overlap with the mean wind speed, was discarded. The wind is an important parameter since it influences animal physiology due to its effects on the cooling factor, which has repercussions on homeothermy, locomotion, and foraging (38). In the case of seabirds, it has been observed that wind speed has an impact on the energetic cost derived from flight (39) or on the strategies used for foraging (40). Taking all this into account, the fact that the maximum gust speed variable did not contribute significantly to the analysis could be explained by the fact that the animals used in this study were in controlled conditions where feed was supplied *ad libitum*. In other animal species such as cattle, wind speed is a key factor in the bovine thermoregulation process, with a marked effect on testicular cooling (41).

After performing the multicollinearity analysis, among the variables that remain, minimum temperature, new moon as moon phase, minimum barometric pressure, rainfall, waxing gibbous and waning crescent as moon phases contribute significantly in determining whether a freezing–thawing ejaculate belongs to one quality group or another.

Minimum temperature showed the highest discriminant power. For the correct development of the reproductive capacity, the roosters must be housed at an adequate ambient temperature. Deviations of 5°C to their thermal optimum result in high reductions in reproductive success (42). Thus, spermatogenesis may be altered by physiological changes resulting from heat stress. For example, in mammals, a reduction in sperm production has been demonstrated because the level of testosterone decreases under heat stress (43). Likewise, too-low temperatures also affect semen quality. In the present research, within the minimum temperatures, the one with the highest value is the one that gives rise to the highest sperm motility. This could be because low temperatures suppress testicular growth and negatively influence fertility in birds, which is ultimately directly related to semen quality (44). Furthermore, several studies are showing how cold decreases testicular development in several bird species (45, 46).

The lunar phase, especially the new moon, also shows a high discriminating power. Birds, during the phases in which luminosity is higher, show a more active behavior reflected in a more powerful song (47, 48). In breeding birds, melatonin concentrations fluctuate according to the lunar rhythm, which is due to the light intensity associated with the different lunar phases (49). Melatonin plays a protective role in testicular development by being a powerful antioxidant that eliminates free radicals in a very effective way. By contrast, in human males, low melatonin levels result in lower sperm quality due to reduced sperm motility (50). In rams and goats, melatonin treatment improves testicular development and seminal quality (51, 52). In any case, according to the results obtained in the present research, when there is a new moon phase, seminal quality decreases. Similar results were obtained in a study carried out on bulls, in which sperm activity was higher when there was a full moon phase compared to data obtained during a new moon phase (53). In contrast, in humans, no significant differences were observed for the sperm motility parameter during the phases of the lunar cycle evaluated (54).

Another variable with high discriminant power is minimum barometric pressure. In the present study, when the minimum barometric pressure is higher, the ejaculate has worse post-thawing motility. This coincides with that reported by Diaz-Usi, Venturina (55) in water buffaloes, in which an increase in barometric pressure has negative effects on sperm motility characteristics, both in fresh semen and after cryopreservation, especially concerning kinematic parameters. This same effect has been observed in bulls (56). On the contrary, in sheep, better seminal quality is observed when the pressure is high since ewes inseminated with sperm obtained in periods when the pressure was low gave birth to lambs with low birth and weaning weights (57). However, the fact that sheep are a short-day seasonal polyestrous species may account for this fact.

Finally, the rainfall variable was also significant in determining the seminal quality group to which a post-thawing ejaculate belongs. Precipitation, among other climatological parameters, is an environmental stress factor that could have an impact on the different physiological processes of the animals (58). However, in goats, during a dry period, a lower percentage of motility and a higher number of abnormal spermatozoa were detected by Van Tilburg, Salles (59). Dry periods correspond to higher temperatures, which could explain the poorer semen quality since elevated temperature is an important source of stress for birds, which respond by modifying organ functions and circulating levels of hormones, glucose, leukocytes, and electrolytes (60, 61). Also, high temperature is associated with lower sperm concentration and motility (5). In addition, heat stress causes avian infertility, which is because high temperatures negatively affect gamete formation and the fertilization process (62). Related to this, sperm exposed to heat has reduced longevity within the uterovaginal junction in hens (63, 64). In addition, in chicken, when the temperature is higher than 32°C and the humidity is at 55–65%, morpho-anomalies in the spermatogenic cells at the testicular level and a decrease in testosterone production can be found (65, 66).

In short, meteorological parameters can influence animal reproduction at different levels such as modulation of the breeding timing, investment, or survival of offspring, as well as originally in spermatogenesis which results in the ejaculate having a certain quality (67–69). The choice of sperm motility as a reference parameter to determine the seminal quality of an ejaculate was due to its importance since this quality-related trait is a reliable predictor of fertility following a freeze–thaw cycle (70, 71), and reflects several aspects of sperm physiology, such as glycolysis, oxidative phosphorylation, and membrane intactness, among others (72). Membrane permeability, lipid composition, and membrane fluidity of the spermatozoa are determining factors in avoiding damage during the cryopreservation process, and the final membrane damage associated with these factors has consequences on sperm motility (8, 73, 74). The fact that sperm motility in freezing–thawing semen is lowered is due to a gradual decrease in energy that reduces the ability of spermatozoa to perform adequate movement to allow fertilization. Thus, ATP production decreases when mitochondrial integrity is impaired, given the high sensitivity of rooster sperm mitochondria to freeze–thaw (11).

Avian spermatozoa have a high sensitivity to high environmental temperatures and temperature changes during cryopreservation. Thus, high-quality ejaculates are necessary before freezing. The assessment of morphometric variables in both fresh and thawed semen can help predict the ability to survive a freeze/thaw cycle since the size of the sperm head determines the permeability of the cell membrane to water and cryoprotectant, which would be directly related to the formation of intracellular ice crystals (75, 76). In this sense, the study of morphometric variables could be a quality criterion to be added in addition to motility to determine the freezability of an ejaculate.

In any case, as suggested by the present work, the choice of the moment of the seminal collection is important since climatic and moon phase-related traits can affect sperm freezability. This could be due to changes in the composition of the sperm at both enzymatic and biochemical levels, as well as to alterations in the hormonal level, which would affect the permeability of the membrane, giving rise to a greater susceptibility to the toxicity generated by cryoprotectants and the rest of the lesions that occur during the cryopreservation process (77–79).

## 5 Conclusion

In conclusion, climatology and the lunar cycle affect sperm freezability. After carrying out a DCA, the minimum temperature, the new moon as moon phase, minimum barometric pressure, rainfall, waxing gibbous, and waning crescent as moon phase’s variables provide a great amount of information and show a high discrimination power when differentiating between sperm quality groups. In rooster species, when the temperature is low, testicular size decreases, leading to a decrease in pre- and post-thaw seminal quality. Variations in melatonin production due to the moon phase were reported to affect semen quality. Thus, less desirable results were found in the new moon phases. This study has developed a tool that will allow us to optimize the work of poultry semen cryopreservation in animal reproduction centers. At present, good results have not been obtained in the cryopreservation of rooster semen. However, the selection of optimal times for semen freezing may represent an advance in the improvement of post-thawing semen quality in this species. Through the conservation of genetic resources of endangered poultry breeds, genetic diversity can be guaranteed. So investing in research for the improvement of germplasm banks will be fundamental for the improvement of reproductive biotechnology in avian species.

## Data availability statement

The original contributions presented in the study are included in the article/supplementary material, further inquiries can be directed to the corresponding author.

## Ethics statement

Ethical approval was not required for the study involving animals in accordance with the local legislation and institutional requirements because the present study is excluded from the scope of evaluation of the Ethics Committee of the University of Cordoba since data obtained are part of routine activities carried out at the Agropecuary Provincial Center of the Diputación of Córdoba (Andalusia, Spain) as a center for avian reproduction and conservation of native breeds and therefore are not considered animals used for scientific purposes. However, all animals used in the study were treated following European Legislation (Directive 2010/63/EU “on the protection of animals used for scientific purposes”), which has been transposed into Spanish law through RD 53/2013.

## Author contributions

ED: Formal analysis, Investigation, Methodology, Writing – original draft, Writing – review & editing. JD: Conceptualization, Funding acquisition, Project administration, Resources, Supervision, Visualization, Writing – review & editing. AGA: Conceptualization, Data curation, Formal analysis, Funding acquisition, Investigation, Methodology, Project administration, Resources, Software, Supervision, Validation, Visualization, Writing – original draft, Writing – review & editing. JL: Data curation, Formal analysis, Funding acquisition, Methodology, Resources, Software, Supervision, Writing – review & editing. AAA: Data curation, Investigation, Methodology, Writing – review & editing. FN: Conceptualization, Data curation, Formal analysis, Methodology, Software, Supervision, Validation, Visualization, Writing – original draft, Writing – review & editing.

## Footnotes

^1^AEMET; http://www.aemet.es/

^2^https://aa.usno.navy.mil/

## References

[1] Shanmugam M Rajkumar U Reddy M Rao SR. Effect of age on semen quality in naked neck and dwarf chicken under tropical climatic conditions. Anim Prod Sci. (2012) 52:964–8. doi: 10.1071/AN12033

[2] Verratti V Di Giulio C D'angeli A Tafuri A Francavilla S Pelliccione F. Sperm forward motility is negatively affected by short-term exposure to altitude hypoxia. Andrologia. (2016) 48:800–6. doi: 10.1111/and.12515, PMID: 26762696

[3] Rao M Zhao X-L Yang J Hu S-F Lei H Xia W. Effect of transient scrotal hyperthermia on sperm parameters, seminal plasma biochemical markers, and oxidative stress in men. Asian J Androl. (2015) 17:668–75. doi: 10.4103/1008-682X.146967, PMID: 25652627 PMC4492061

[4] Longobardi V Zullo G Salzano A De Canditiis C Cammarano A De Luise L. Resveratrol prevents capacitation-like changes and improves in vitro fertilizing capability of buffalo frozen-thawed sperm. Theriogenology. (2017) 88:1–8. doi: 10.1016/j.theriogenology.2016.09.046, PMID: 27865407

[5] Vandana G Sejian V Lees A Pragna P Silpa M Maloney SK. Heat stress and poultry production: impact and amelioration. Int J Biometeorol. (2021) 65:163–79. doi: 10.1007/s00484-020-02023-7, PMID: 33025116

[6] Zimecki M. The lunar cycle: effects on human and animal behavior and physiology. Postepy Hig Med Dosw. (2006) 60:1–7. 16407788

[7] Mocé E Graham JK. In vitro evaluation of sperm quality. Anim Reprod Sci. (2008) 105:104–18. doi: 10.1016/j.anireprosci.2007.11.016 18178345

[8] Long J. Avian semen cryopreservation: what are the biological challenges? Poult Sci. (2006) 85:232–6. doi: 10.1093/ps/85.2.232, PMID: 16523619

[9] Santiago-Moreno J Castaño C Toledano-Díaz A Coloma M López-Sebastián A Prieto M. Semen cryopreservation for the creation of a Spanish poultry breeds cryobank: optimization of freezing rate and equilibration time. Poult Sci. (2011) 90:2047–53. doi: 10.3382/ps.2011-01355, PMID: 21844272

[10] Blesbois E. Freezing avian semen. Avian Biol Res. (2011) 4:52–8. doi: 10.3184/175815511X13069413108523

[11] Santiago-Moreno J Castaño C Toledano-Díaz A Coloma M López-Sebastián A Prieto M. Influence of season on the freezability of free-range poultry semen. Reprod Domest Anim. (2012) 47:578–83. doi: 10.1111/j.1439-0531.2011.01921.x, PMID: 21988546

[12] MAPA. *Animal germplasm banks*. (2024). Available at: https://www.mapa.gob.es/es/ganaderia/temas/zootecnia/razas-ganaderas/bancos-germoplasma/.

[13] Churchil RR Praveena PE Sharma D. Semen quality parameters, their inter-relationship and post-washing sperm attributes of Rhode Island red roosters. Vet. World. (2014) 7:1117–22. doi: 10.14202/vetworld.2014.1117-1122

[14] Gandini G Oldenbroek K. Strategies for moving from conservation to utilisation In: Oldenbroek K, editor. Utilisation and conservation of farm animal genetic resources. Wageningen, Netherlands: Wageningen Academic Publishers (2007). 29–54.

[15] Campo J. *Razas de gallinas autóctonas andaluzas: Andaluza Azul, Andaluza Utrerana y Combatiente Español*. Patrimonio ganadero andaluz: las razas ganaderas de Andalucía (Volumen II); Consejería de Agricultura y Pesca. (2007).

[16] Barba C Fernández-Tomillo L Jiménez R Guzmán J García A. Environmental ecological value and conservation of local sheep breeds endangered in Andalusia. Arch. Zootec. (2016) 65:445–8.

[17] González Ariza A Arando Arbulu A León Jurado JM Navas González FJ Delgado Bermejo JV Camacho Vallejo ME. Discriminant canonical tool for differential biometric characterization of multivariety endangered hen breeds. Animals. (2021) 11:2211. doi: 10.3390/ani11082211, PMID: 34438669 PMC8388411

[18] Burrows W Quinn J. The collection of spermatozoa from the domestic fowl and Turkey. Poult Sci. (1937) 16:19–24. doi: 10.3382/ps.0160019

[19] Sasaki K Tatsumi T Tsutsui M Niinomi T Imai T Naito M. A method for cryopreserving semen from Yakido roosters using N-methylacetamide as a cryoprotective agent. J Poult Sci. (2010) 47:297–301. doi: 10.2141/jpsa.009111

[20] Boes J Boettcher P Honkatukia M. Innovations in cryoconservation of animal genetic resources: practical guide. Rome, Italy: Food and Agriculture Organization (2023).

[21] Poulsen J French A. Discriminant function analysis. San Francisco, CA: San Francisco State University (2008).

[22] Navas CM Bermejo JVD McLean AK Jurado JML González FJN. Discriminant canonical analysis of the contribution of Spanish and Arabian purebred horses to the genetic diversity and population structure of Hispano-Arabian horses. Animals. (2021) 11:269. doi: 10.3390/ani11020269 33494478 PMC7912545

[23] Rogerson PA. Data reduction: factor analysis and cluster analysis. Stat Methods Geogr. (2001) 2001:192–7. doi: 10.4135/9781849209953.n10

[24] Zhang Q Hu J Bai Z. Modified Pillai’s trace statistics for two high-dimensional sample covariance matrices. J Stat Plan Inference. (2020) 207:255–75. doi: 10.1016/j.jspi.2020.01.002

[25] Anuthama K Shankar S Ilayaraja V Kumar GS Rajmohan M. Determining dental sex dimorphism in south Indians using discriminant function analysis. Forensic Sci Int. (2011) 212:86–9. doi: 10.1016/j.forsciint.2011.05.018, PMID: 21664775

[26] González Ariza A Arando Arbulu A Navas González FJ Delgado Bermejo JV Camacho Vallejo ME. Discriminant canonical analysis as a validation tool for multivariety native breed egg commercial quality classification. Food Secur. (2021) 10:632. doi: 10.3390/foods10030632, PMID: 33802707 PMC8002516

[27] Toalombo Vargas PA Navas González FJ Landi V León Jurado JM Delgado Bermejo JV. Sexual dimorphism and breed characterization of creole hens through biometric canonical discriminant analysis across Ecuadorian agroecological areas. Animals. (2019) 10:32. doi: 10.3390/ani10010032 31877907 PMC7022707

[28] Pugh DT. *Tides, surges and mean sea level* (1987).

[29] Le Blancq F. Diurnal pressure variation: the atmospheric tide. Weather. (2011) 66:306–7. doi: 10.1002/wea.857

[30] Neild R. Maximum-minimum temperatures as a basis for evaluating thermoperiodic response. Mon Weather Rev. (1967) 95:583–4. doi: 10.1175/1520-0493(1967)095<0583:MMTAAB>2.3.CO;2

[31] Subramaniam K Padalkar S. Visualisation and reasoning in explaining the phases of the moon. Int J Sci Educ. (2009) 31:395–417. doi: 10.1080/09500690802595805

[32] Das S Dodd S Lewis-Jones DI Patel FM Drakeley AJ Kingsland CR. Do lunar phases affect conception rates in assisted reproduction? J Assist Reprod Genet. (2005) 22:15–8. doi: 10.1007/s10815-005-0815-y, PMID: 15807217 PMC3455390

[33] Ellis HI Jehl JR Jr. Total body water and body composition in phalaropes and other birds. Physiol Zool. (1991) 64:973–84. doi: 10.1086/physzool.64.4.30157952

[34] Nishimura T Fukushima M. Why animals respond to the full moon: magnetic hypothesis. Biosci Hypotheses. (2009) 2:399–401. doi: 10.1016/j.bihy.2009.06.006

[35] Yonezawa T Uchida M Tomioka M Matsuki N. Lunar cycle influences spontaneous delivery in cows. PLoS One. (2016) 11:e0161735. doi: 10.1371/journal.pone.0161735, PMID: 27580019 PMC5006988

[36] Chinchilla-Vargas J Kerns K Rothschild MF. Lunar and climatic effects on boar ejaculate traits. Anim Reprod Sci. (2018) 193:117–25. doi: 10.1016/j.anireprosci.2018.04.006, PMID: 29661543

[37] Kollerstrom N. Lunar effect on thoroughbred mare fertility: an analysis of 14 years of data, 1986–1999. Biol Rhythm Res. (2004) 35:317–27. doi: 10.1080/0929-1010400000624

[38] Møller A. Long-term trends in wind speed, insect abundance and ecology of an insectivorous bird. Ecosphere. (2013) 4:1–11. doi: 10.1890/ES12-00310.1

[39] Amélineau F Péron C Lescroël A Authier M Provost P Grémillet D. Windscape and tortuosity shape the flight costs of northern gannets. J Exp Biol. (2014) 217:876–85. doi: 10.1242/jeb.097915, PMID: 24622894

[40] Dehnhard N Ludynia K Poisbleau M Demongin L Quillfeldt P. Good days, bad days: wind as a driver of foraging success in a flightless seabird, the southern rockhopper penguin. PLoS One. (2013) 8:e79487. doi: 10.1371/journal.pone.0079487, PMID: 24236139 PMC3827366

[41] Overton M Sischo W Temple G Moore D. Using time-lapse video photography to assess dairy cattle lying behavior in a free-stall barn. J Dairy Sci. (2002) 85:2407–13. doi: 10.3168/jds.S0022-0302(02)74323-3, PMID: 12362476

[42] Schou MF Bonato M Engelbrecht A Brand Z Svensson EI Melgar J. Extreme temperatures compromise male and female fertility in a large desert bird. Nat Commun. (2021) 12:666. doi: 10.1038/s41467-021-20937-7, PMID: 33531493 PMC7854745

[43] Moreno RD Lagos-Cabré R Buñay J Urzúa N Bustamante-Marín X. Molecular basis of heat stress damage in mammalian testis. Testis. (2012):127–55.

[44] Huston TM. The effects of environmental temperature on fertility of the domestic fowl. Poult Sci. (1975) 54:1180–4. doi: 10.3382/ps.0541180 1161704

[45] Perfito N Tramontin AD Meddle S Sharp P Afik D Gee J. Reproductive development according to elevation in a seasonally breeding male songbird. Oecologia. (2004) 140:201–10. doi: 10.1007/s00442-004-1576-5 15148599

[46] Silverin B Wingfield J Stokkan K-A Massa R Järvinen A Andersson N-Å. Ambient temperature effects on photo induced gonadal cycles and hormonal secretion patterns in great tits from three different breeding latitudes. Horm Behav. (2008) 54:60–8. doi: 10.1016/j.yhbeh.2008.01.015, PMID: 18402961

[47] York JE Young AJ Radford AN. Singing in the moonlight: dawn song performance of a diurnal bird varies with lunar phase. Biol Lett. (2014) 10:20130970. doi: 10.1098/rsbl.2013.0970, PMID: 24429683 PMC3917340

[48] Andrade RM Rojas JA Espinoza MM Viamonte KR. *Influencia lunar en cultivos, animales y ser humano*. Uniandes Episteme Revista de Ciencia, Tecnología e Innovación, no. 4, pp. 37–47. (2017).

[49] Tarlow EM Hau M Anderson DJ Wikelski M. Diel changes in plasma melatonin and corticosterone concentrations in tropical Nazca boobies (*Sula granti*) in relation to moon phase and age. Gen Comp Endocrinol. (2003) 133:297–304. doi: 10.1016/S0016-6480(03)00192-8 12957473

[50] Awad H Halawa F Mostafa T Atta H. Melatonin hormone profile in infertile males. Int J Androl. (2006) 29:409–13. doi: 10.1111/j.1365-2605.2005.00624.x, PMID: 16371109

[51] Ramadan T Taha T Samak M Hassan A. Effectiveness of exposure to longday followed by melatonin treatment on semen characteristics of Damascus male goats during breeding and non-breeding seasons. Theriogenology. (2009) 71:458–68. doi: 10.1016/j.theriogenology.2008.07.029, PMID: 18817966

[52] Casao A Vega S Palacín I Pérez-Pe R Laviña A Quintín F. Effects of melatonin implants during non-breeding season on sperm motility and reproductive parameters in rasa Aragonesa rams. Reprod Domest Anim. (2010) 45:425–32. doi: 10.1111/j.1439-0531.2008.01215.x 18954380

[53] Shestakov V Ermoshina E Korolev V Pimkina T Koroleva E. Variability of the generative function of bulls of different breeds and their tolerance in connection with the change in the lunar phases In: Shestakov V, editor. IOP conference series: Earth and environmental science. Bristol, UK: IOP Publishing (2021)

[54] Moretti E Tallis V Trovarelli S Gnech M Capitani S Ponchietti R. Do lunar phases influence semen parameters. J Androl Sci. (2008) 15:158–63.

[55] Diaz-Usi J Venturina E Peralta M Duran P Mingala C Medina N.. Effect of weekly changes in environmental parameters on sperm motility characteristics of water buffalo (*Bubalus bubalis* Linn.) bulls. AIP Conf Proc, (2023), 2628: 070010. doi: 10.1063/5.0145393

[56] Abilov A Amerhanov CA Korneyenko-Zhilyaev YA Pyzhova E Kombarova N Vinogradova I. Effect of atmospheric pressure on semen parameters in bull sires of modern selection on the day of collection. Agric Biol. (2017) 52:314–22. doi: 10.15389/agrobiology.2017.2.314eng

[57] Malikov D. *The effect of atmospheric pressure on reproduction in rams* (1963).

[58] Abecia J Arrébola F Macías A Laviña A González-Casquet O Benítez F. Temperature and rainfall are related to fertility rate after spring artificial insemination in small ruminants. Int J Biometeorol. (2016) 60:1603–9. doi: 10.1007/s00484-016-1150-y, PMID: 26951115

[59] Van Tilburg M Salles M Silva M Moreira R Moreno F Monteiro-Moreira A. Semen variables and sperm membrane protein profile of Saanen bucks (*Capra hircus*) in dry and rainy seasons of the northeastern Brazil (3 S). Int J Biometeorol. (2015) 59:561–73. doi: 10.1007/s00484-014-0869-6, PMID: 25086569

[60] Smith EM Blalock JE. A complete regulatory loop between the immune and neuroendocrine systems operates through common signal molecules (hormones) and receptors. Fed Proc. (1985) 44:108–11. doi: 10.1007/978-1-4899-0557-4_11 2578415

[61] Mitchell M Kettlewell P. Physiological stress and welfare of broiler chickens in transit: solutions not problems! Poult Sci. (1998) 77:1803–14. doi: 10.1093/ps/77.12.1803, PMID: 9872583

[62] Mcdaniel CD Bramwell RK Wilson JL Howarth B Jr. Fertility of male and female broiler breeders following exposure to elevated ambient temperatures. Poult Sci. (1995) 74:1029–38. doi: 10.3382/ps.0741029, PMID: 7644414

[63] Mcdaniel CD Bramwell RK Howarth JRB. The male contribution to broiler breeder heat-induced infertility as determined by sperm-egg penetration and sperm storage within the hen's oviduct. Poult Sci. (1996) 75:1546–54. doi: 10.3382/ps.0751546, PMID: 9000282

[64] King L Brillard J Garrett W Bakst M Donoghue A. Segregation of spermatozoa within sperm storage tubules of fowl and Turkey hens. Reproduction. (2002) 123:79–86. doi: 10.1530/rep.0.1230079, PMID: 11869189

[65] Chen Z Zhang J Zhou Y Liang C Jiang Y. Effect of heat stress on the pituitary and testicular development of Wenchang chicks. Arch Anim Breed. (2015) 58:373–8. doi: 10.5194/aab-58-373-2015

[66] Xiong Y Yin Q Li J He S. Oxidative stress and endoplasmic reticulum stress are involved in the protective effect of alpha lipoic acid against heat damage in chicken testes. Animals. (2020) 10:384. doi: 10.3390/ani10030384, PMID: 32120945 PMC7142828

[67] Møller AP Fiedler W Berthold P. Effects of climate change on birds. Oxford, UK: OUP Oxford (2010).

[68] Visser ME Both C Lambrechts MM. Global climate change leads to mistimed avian reproduction. Adv Ecol Res. (2004) 35:89–110. doi: 10.1016/S0065-2504(04)35005-1

[69] Karaca A Parker H McDaniel C. Elevated body temperature directly contributes to heat stress infertility of broiler breeder males. Poult Sci. (2002) 81:1892–7. doi: 10.1093/ps/81.12.1892, PMID: 12512583

[70] Nguyen TMD. Main signaling pathways involved in the control of fowl sperm motility. Poult Sci. (2019) 98:1528–38. doi: 10.3382/ps/pey465, PMID: 30476283

[71] Blesbois E Grasseau I Seigneurin F Mignon-Grasteau S Saint Jalme M Mialon-Richard M. Predictors of success of semen cryopreservation in chickens. Theriogenology. (2008) 69:252–61. doi: 10.1016/j.theriogenology.2007.09.019, PMID: 17977587

[72] Van der Horst G. Status of sperm functionality assessment in wildlife species: from fish to primates. Animals. (2021) 11:1491. doi: 10.3390/ani11061491, PMID: 34064087 PMC8224341

[73] Lake P. *The male in reproduction*. (1984).

[74] Blesbois E. Membrane fluidity and the ability to survive cryopreservation in domestic bird spermatozoa. Reproduction. (2005) 129:371–8. doi: 10.1530/rep.1.00454, PMID: 15749963

[75] Esteso MC Soler AJ Fernández-Santos MR Quintero-Moreno AA Garde JJ. Functional significance of the sperm head morphometric size and shape for determining freezability in Iberian red deer (*Cervus elaphus hispanicus*) epididymal sperm samples. J Androl. (2006) 27:662–70. doi: 10.2164/jandrol.106.000489 16728722

[76] Peña FJ Saravia F García-Herreros M Núñezmartínez I Tapia JA Johannisson A. Identification of sperm morphometric subpopulations in two different portions of the boar ejaculate and its relation to postthaw quality. J Androl. (2005) 26:716–23. doi: 10.2164/jandrol.05030, PMID: 16291966

[77] Bentley L Ansah G Buckland R. Seminal plasma proteins of a line of chickens selected for fertility of frozen-thawed semen and the control line. Poult Sci. (1984) 63:1444–5. doi: 10.3382/ps.0631444, PMID: 6473258

[78] Blesbois E De Reviers M. Effect of different fractions of seminal plasma on the fertilizing ability of fowl spermatozoa stored in vitro. Reproduction. (1992) 95:263–8. doi: 10.1530/jrf.0.0950263, PMID: 1625241

[79] Blesbois E Brillard J-P. Specific features of in vivo and in vitro sperm storage in birds. Animal. (2007) 1:1472–81. doi: 10.1017/S175173110700081X 22444920

